# Development of a Modified Intimate Wash From Yoniprakshalan Kwatha (YOSHA) With Comparative Antimicrobial and Quality Assessment: Protocol for an Experimental Study

**DOI:** 10.2196/73239

**Published:** 2026-04-17

**Authors:** Prachi Vandan Jadhav, Bharat Jagdishji Rathi

**Affiliations:** 1 Department of Rasashashtra and Bhaishajya Kalpana, Mahatma Gandhi Ayurved College and Research Centre, Datta Meghe Institute of Higher Education and Research Centre, Paloti Road, Sawangi Meghe, Wardha, 442001, India, 91 7058795090 ext 9011058301

**Keywords:** yoniprakshalan kwatha, yoniwash, YOSHA, antimicrobial, Ayurveda

## Abstract

**Background:**

Vaginal itching is a frequent gynecological issue impacting women of various age groups. Although commonly linked to nonserious conditions, it may also signal infections, sexually transmitted diseases, prolonged use of certain medications, such as oral contraceptives, hormonal fluctuations, or suboptimal hygiene. Standard pharmaceutical treatments, despite being widely used, often produce undesirable effects, such as irritation of the vaginal lining, alterations in vaginal pH, and disruption of healthy microbial flora. These challenges highlight the need for safer, natural alternatives rooted in traditional systems of medicine, such as Ayurveda.

**Objective:**

This study aims to prepare *yoniprakshalan kwatha* (intimate wash decoction) as per the classical reference of *Yogratnakar* and to develop its modified dosage form, YOSHA. Additionally, this study aims to assess the quality control parameters of both yoniprakshalan kwatha and YOSHA to ensure their safety and efficacy. Furthermore, the antimicrobial effects of both formulations will be investigated, and a comparative analysis will be conducted to evaluate and contrast the antimicrobial activity of YOSHA and yoniprakshalan kwatha.

**Methods:**

Medicinal herbs were sourced from the institute’s *rasashala* and authenticated by taxonomists. The traditional decoction and YOSHA will be prepared using Ayurvedic and modern pharmaceutical methods. Quality tests will include physicochemical and microbial analyses. The disc diffusion method will be used to evaluate antimicrobial activity against *Candida albicans, Escherichia coli,* and *Staphylococcus aureus*. Standard antibiotics and sterile discs will serve as controls. Tests will be repeated over 3 days. Statistical analysis will be performed using one-way ANOVA in SPSS (version 17), with *P*<.05 considered significant.

**Results:**

This study commenced in July 2025, with data collection scheduled from August 2025 to September 2025. By December 2025, it is expected that laboratory testing and antimicrobial assessments will have been completed, along with the finalization of quality control evaluations. Preliminary findings are anticipated to show that YOSHA will exhibit strong antimicrobial activity, comparable to or exceeding that of the original yoniprakshalan kwatha. Significant inhibition zones are likely to be observed against *C albicans* and *E coli*. The modified formulation is projected to meet pharmaceutical standards for consistency, safety, and shelf stability. Data analysis will be completed, and the full results are expected to be published in December 2025.

**Conclusions:**

The study will show that YOSHA, a modern herbal product based on the traditional Ayurvedic yoniprakshalan kwatha, has strong in vitro antimicrobial effects against common vaginal pathogens and meets pharmaceutical quality and safety standards. Unlike raw decoctions, it offers a stable, user-friendly formulation that blends traditional knowledge with scientific validation. Strengths include classical preparation methods, solid quality control, and reliable results. Future work should involve clinical trials, broader testing, and user-focused evaluations. Findings will be disseminated through journals, conferences, and practitioner outreach to promote YOSHA as a natural option for women’s hygiene.

## Introduction

### Background

Vaginal itching is a common and typically harmless condition that most women experience at some point in their lives. It can be triggered by a variety of factors, including infections, hormonal changes, sexually transmitted diseases, prolonged use of medications such as birth control pills, or poor vaginal hygiene. Additionally, certain skin conditions, such as psoriasis, eczema, or a vaginal yeast infection, can contribute to the irritation. In rare cases, it may be a symptom of a more serious underlying condition, such as vulvar cancer. If left untreated, vaginal itching can potentially lead to serious complications or, in extreme cases, become life-threatening [1].

Treating these conditions with oral medications is as important as topical therapies [2]. Female genital disorders are treated using antibiotics, antifungals, antiprotozoals, and other medications administered both orally and topically. The *sthanik chikitsa* (local treatment) recommended by the ancient Acharyas has shown excellent and satisfactory results in the management of *stree roga* (gynecological disorders). These localized therapies are particularly effective in treating *tryavarta yoni* disease [3]. According to the World Health Organization, an estimated 333 million new cases of treatable vulvovaginal infections occur globally each year. A study conducted in India reported a 37% prevalence of reproductive tract infections based on symptoms and a 36.7% prevalence based on laboratory findings. Among these cases, 31% were due to candidiasis, 3% to gonorrhea, 2% to trichomoniasis, and 45% to bacterial vaginosis [4].

Using intimate wash is important for maintaining vaginal health. It helps preserve the natural pH balance of the vagina, which is essential for preventing infections and discomfort. Practicing proper intimate hygiene can significantly reduce the risk of bacterial and fungal infections, including yeast infections and bacterial vaginosis [5]. Using a gentle, specially formulated wash for the intimate area can help eliminate odor-causing bacteria and keep the area feeling clean and refreshed [6]. Additionally, regular use of an intimate wash can enhance overall comfort and confidence, helping women feel clean and hygienic throughout the day. In essence, a female intimate wash plays a vital role in supporting women’s reproductive health and overall well-being [7].

*Yoniprakshalana kwatha* (intimate wash decoction) [8] is the formulation described in the *Yogratnakar* for the treatment of *yonikandu* and includes *triphala, gudhuchi*, and *dantimul*. In this study, this traditional *kwatha* will be adapted into a ready-to-use surfactant-based vaginal wash, named YOSHA. The term “YOSHA” signifies “woman” (*stree*) [9]. The name “YOSHA” is derived from the words *yoni* (“YO”) and *wash* (“SH”), symbolizing a product designed specifically for cleansing the *yoni* (vagina) and intended for women. This modified version of a medicated intimate wash, enriched with the benefits of traditional herbs, offers a more convenient and travel-friendly alternative to the freshly prepared *kwatha* (decoction). Compared with the original decoction, the improved formulation ensures easier use, thoroughly cleanses the vaginal area, and provides a refreshing, hygienic effect. This intimate wash is based on the classical formulation for *yoniprakshalan kwatha* described in ancient Ayurvedic texts, particularly the *Yogratnakar*. This study aims to formulate a modification of yoniprakshalan kwatha as an intimate wash (YOSHA) and to conduct a comparative quality control assessment and in vitro evaluation of antimicrobial activity.

### Need for the Study

Maintaining vaginal hygiene is essential, especially in today’s fast-paced world, where working women and school-going girls are frequently exposed to public restrooms. While there are several intimate wash products available on the market, many contain harsh chemicals that can lead to side effects such as dryness and itching.

To address this, we have developed a simplified and user-friendly version of *yonidhavan kwatha*. Unlike the traditional formulation, which has a short shelf life of about 24 hours, YOSHA is designed in a surfactant-based form that is both effective and convenient. It helps maintain healthy vaginal pH and ensures thorough daily cleansing.

YOSHA is travel friendly, easy to handle, and simple to use, eliminating the need for complex preparation. By offering this product, we support women in maintaining their intimate health and well-being with comfort and confidence.

This study aims to formulate a modification of yoniprakshalan kwatha as an intimate wash (YOSHA) and to conduct a comparative quality control assessment and in vitro evaluation of antimicrobial activity.

### Objectives

The objective of this study is to prepare yoniprakshalan kwatha as per the classical reference of *Yogratnakar* and to develop its modified dosage form, YOSHA. Additionally, the study aims to assess the quality control parameters of both yoniprakshalan kwatha and YOSHA to ensure their safety and efficacy. Furthermore, the antimicrobial effects of both formulations will be investigated, with a comparative analysis conducted to evaluate and contrast the antimicrobial activity of YOSHA and yoniprakshalan kwatha.

## Methods

### Drug Collection and Authentication

An in vitro experimental study will be conducted at the Central Microbiology Laboratory of Jawaharlal Nehru Medical College, a constituent of the Datta Meghe Institute of Higher Education and Research, located in Sawangi (Meghe), Wardha, Maharashtra. The study will be conducted between July 2025 and December 2025.

The research drugs will be procured from Vedpoorna Ayurveda Rasashala, Mahatma Gandhi Ayurved College Hospital and Research Centre (MGACHRC), Salod (H), Wardha. The Dravyaguna Department at MGACHRC will be responsible for the authentication and verification of the raw drugs. These raw materials will be standardized according to the Ayurvedic Pharmacopoeia of India guidelines. Necessary studies will be conducted at certified or recognized institutes, organizations, or laboratories of national repute, as endorsed or recommended by the Datta Meghe Institute of Higher Education and Research. An experimental study will be conducted at the Department of Rasashastra and Bhaishajya Kalpana, MGACHRC, from July 2025 to December 2025.

### Data Handling

The data management plan will follow strict protocols for accurate entry, coding, security, and storage to ensure data integrity. Standardized methods, including double entry for key data and range checks, will minimize errors. Data will be coded according to established guidelines to support analysis. Security measures such as restricted access, encryption, and secure storage (both electronic and physical) will be implemented. Regular backups will prevent data loss, and all procedures will be detailed in the study protocol for transparency.

### Drug Review

Yoniprakshalan kwatha (intimate wash decoction) is an important herbo-mineral formulation described in a book by Vaidya Lakshmpati Sastri, published by Chaukhamba Prakashan, Varanasi, with a Hindi commentary by Bhisagratna and Brahmasankar Sastri. This formulation holds significant therapeutic value and contains ingredients such as *guduchi, amalaki, haritaki*, *bibhitaki*, and (Table 1) *dantimul*. It is primarily indicated for the treatment of *yoni kandu* (vaginal itching).

**Table 1.** Ingredients of *yoniprakshalan kwatha* along with their properties.

Ingredient	Botanical name	Family	Property and description
Amalaki [10]	*Emblica officinalis* Gaertn	Phyllanthaceae	Rasa: Pancharasa (lavanarasa rahita), amlaka pradhana Virya: sheeta Vipak: madhur Part used: fruit Quantity: 1 part
Haritaki [11]	*Terminalia chebula* Retz	Combretaceae	Rasa: Pancharasa (lavan varjita), kashaya pradhana Virya: ushna Vipak: madhur Part used: fruit Quantity: 1 part
Bibhitak [11]	*Terminalia bellirica* Roxb	Combretaceae	Rasa: kashaya Virya: ushna Vipak: madhur Part used: fruit Quantity: 1 part
Guduchi [11]	*Tinospora cordifolia* Miers	Menispermaceae	Rasa: tikta, kashaya Virya: ushna Vipak: madhur Part used: stem Quantity: 1 part
Danti [11]	*Baliospermum montanum* Muell-Arg	Euphorbiaceae	Rasa: katu Virya: ushna Vipak: root Quantity: 1 part

### Detail of Drug Preparation

In the first step, yoniprakshalan kwatha will be prepared according to the textual reference and in the second step, its modified dosage form, YOSHA, will be prepared as mentioned in the following sections (Table 2).

**Table 2.** Ingredients and compositions of yoniprakshalan kwatha (intimate wash decoction).

Ingredients	Botanical name	Part used	Proportion
Amalaki	*Emblica officinalis* Gaertn.	Fruit	1 part
Haritaki	*Terminalia chebula* Retz	Fruit	1 part
Bibhitaki	*Terminalia bellirica* Roxb	Fruit	1 part
Guduchi	*Tinospora cordifolia* Miers	Stem	1 part
Dantimoola	*Baliospermum montanum* Muell-Arg.	Root	1 part
Water	—^a^	—	16 parts of total drugs

a Not available.

#### Preparation of Yoniprakshalan Kwatha

Yoniprakshalan kwatha will be prepared according to the classical method described in the *Yogratnakar* [12], using authenticated raw materials and standard *kwatha* preparation procedures (Textbox 1).

Textbox 1. **Preparation step of yoniprakshalan kwatha.**

**Step 1: inspection of raw drugs**

- Remove deformed pieces or contaminants

**Step 2: powdering and mixing**

- Take all raw drugs in coarse powder form
- Use a clean stainless-steel vessel

**Step 3: soaking**

- Add 16 parts of water to the mixture
- Leave undisturbed for 12 hours

**Step 4: boiling**

- Boil the mixture
- Reduce it to 1/8th of the original volume

**Step 5: filtration**

- Filter the decoction using a clean cloth

**Step 6: final product**

- The filtrate is yoniprakshalana kwatha

#### Preparation of YOSHA

The modified dosage form, YOSHA, will be prepared from yoniprakshalan kwatha using a surfactant-based formulation approach to obtain a stable, ready-to-use intimate wash (Table 3 and Textbox 2).

**Table 3.** Composition of YOSHA.

Ingredients	Percentage	Weight
Yoniprakshalan kwatha (intimate wash decoction)	w/w 40	w/w 40 mL
Methylparaben (preservative) [13]	w/w 4	w/w 4 gm
Sodium metabisulfite (antioxidant)	w/w 4	w/w 4 gm
Hydroxypropyl methylcellulose [14]	w/w 8	w/w 8 gm
Tartaric acid [15]	w/w 4	w/w 4 gm
Distilled water	w/w 40	w/w 40 mL
Total	100	100 ml w/w YOSHA

Textbox 2. **Preparation steps of YOSHA.**

**Step 1: base decoction preparation**

- Take yoniprakshalana kwatha in a clean vessel

**Step 2: pH balancing**

- Add tartaric acid to adjust pH

**Step 3: preservation and antioxidation**

- Add methylparaben (preservative)
- Add sodium metabisulfite (antioxidant)

**Step 4: viscosity adjustment**

- Dissolve hydroxypropyl methylcellulose in distilled water
- Add the hydroxypropyl methylcellulose gel base to the decoction

**Step 5: final mixing and storage**

- Stir thoroughly to ensure uniformity
- Store in an airtight glass container

### Antimicrobial Study

This study will evaluate the antimicrobial activity of yoniprakshalan kwatha and YOSHA using the agar well diffusion method [16].

#### Principle of Antimicrobial Study

A total of 9 agar plates per treatment will be used, and the antimicrobial test will be repeated 3 times. The antimicrobial constituents in yoniprakshalan kwatha and YOSHA will be allowed to diffuse into the agar medium and interact with a plate freshly inoculated with the test organism. The resulting inhibition zone will form a uniform circle, as the growth will spread continuously. The diameter of the inhibition zone can be measured in millimeters. The agar well will be 10 mm in diameter and will hold around 0.5 mL—this volume fills the well evenly, ensuring full contact with the agar surface and avoiding overflows that could interfere with diffusion. Yoniprakshalan kwatha and YOSHA will be introduced into the wells, and ofloxacin 10 μg/mL will be used as the standard for the study. The petri dishes will then be incubated at 37 °C for 24 hours, and the zones of inhibition will be measured [17].

#### Selection of Test Microorganisms

The bacterial strains for the experiment will be obtained from the microbiology department of a reputable institute [18]. The antimicrobial activity of the formulations will be assessed against selected gram-positive bacteria, gram-negative bacteria, and fungal strains commonly associated with vaginal infections. The gram-positive bacteria tested will include *Streptococcus aureus* (National Commission for Indian Systems of Medicine [NCISM] 2079) [19] and *Streptococcus epidermidis* (NCISM 2493) [20]. The gram-negative bacteria evaluated will include *Escherichia coli* (NCISM 2931) [21] and *Proteus vulgaris* (NCISM 2027) [22]. The fungal strain tested will be *Candida albicans* [23]*.*

#### Positive Control Group Drugs

Standard antimicrobial agents will be used as positive controls to validate the antimicrobial assay. For gram-positive bacteria, azithromycin (50 µg/6 µL) will be used. For gram-negative bacteria, ciprofloxacin (30 µg/6 µL) will be used. For *C albicans*, clotrimazole (50 µg/6 µL) will be used.

### Analytical Study of Drugs

#### Analytical Parameters of Yoniprakshalan Kwatha

The analytical evaluation of yoniprakshalan kwatha will include the assessment of organoleptic and physicochemical parameters. Organoleptic characteristics, including touch, appearance, taste, and odor, will be examined [24]. Physicochemical parameters will include alcohol content, pH value, specific gravity at 25 °C, total solids, water-soluble extractive values, alcohol-soluble extractive values, and high-performance thin-layer chromatography [25].

#### Analytic Parameter for Yoni Wash (YOSHA)

YOSHA will be evaluated using organoleptic and physicochemical parameters. Organoleptic characteristics, including touch, appearance, and odor, will be assessed [26]. Physicochemical evaluation will include pH, water-soluble extractive values or alcohol-soluble extractive values, and tests for specific pathogens [27].

#### Data Analysis

The data will be analyzed to determine the effects of the test compounds on the assessed parameters. This will typically involve statistical analysis to compare the treated groups to the control group.

#### Summary

Pharmaceutical and analytical studies of yoniprakshalan kwatha and its modified form as intimate wash will provide the standard parameters.

#### Study Guidelines

This manuscript will adhere to the Checklist for Reporting In-vitro Studies (CRIS) guidelines [28] to ensure methodological transparency and comprehensive reporting of in vitro research. The CRIS guidelines were followed during the preparation and reporting of this study.

### Statistical Analysis

Data analysis will be conducted using SPSS (version 17; IBM Corp). Three groups—YOSHA, yoniprakshalan kwatha, and a standard antimicrobial control—will be compared, with a minimum of 9 replicates per group. Descriptive statistics will include means (SD) for continuous variables and percentages for categorical data. Normality and homogeneity of variance will be assessed using the Shapiro-Wilk test and the Levene test, respectively. One-way ANOVA will be applied to compare antimicrobial activity (zone of inhibition), followed by the Tukey honestly significant difference post hoc test if significant differences are found (*P*<.05). If ANOVA assumptions are violated, nonparametric alternatives such as the Kruskal-Wallis test will be used. Regression analysis will be performed if dose-response data are available. This approach ensures statistical validity and reliable comparisons between formulations.

### Ethical Considerations

Ethical approval for the research has been granted by the Institutional Ethics Committee (MGACHRC/IEC/Jun-2024/854) of the Datta Meghe Institute of Higher Education & Research, Salod (H), Wardha-442004, Maharashtra.

## Results

This study commenced in July 2025, with data collection scheduled from August 2025 to September 2025. By December 2025, it is expected that laboratory testing and antimicrobial assessments will have been completed, along with the finalization of quality control evaluations. Preliminary findings are anticipated to show that YOSHA will exhibit strong antimicrobial activity, comparable to or exceeding that of the original yoniprakshalan kwatha. Significant inhibition zones are likely to be observed against *C albicans* and *E coli*. The modified formulation is projected to meet pharmaceutical standards for consistency, safety, and shelf stability. Data analysis will be completed, and full results are expected to be published in December 2025.

## Discussion

### Anticipated Findings

This study is expected to demonstrate that YOSHA, a modified formulation of yoniprakshalan kwatha, will demonstrate strong in vitro antimicrobial activity, particularly against *C albicans* and *E coli*. Designed as a modern, surfactant-based intimate wash, YOSHA is expected to meet the pharmaceutical standards of stability, safety, and usability while preserving Ayurvedic therapeutic principles. The aim is to convert a traditional decoction into a stable, ready-to-use product with improved shelf life and consistent quality. YOSHA may offer a safer, more convenient alternative to commercial feminine hygiene products, many of which can disrupt vaginal pH or flora [29].

While Ayurvedic treatments, such as yoniprakshalan kwatha, are traditionally used for vaginal conditions such as *yoni kandu* [30], their practical application is limited by preparation complexity and short shelf life [31]. YOSHA addresses these gaps through standardized pharmaceutical development. Although the individual herbs used (*triphala, guduchi,* and *dantimul*) have known antimicrobial properties [32], their combined and stabilized use in topical form remains underexplored. The strengths of this study lie in its grounding in classical texts, the use of validated preparation methods, and laboratory testing. However, as an in vitro study, it lacks clinical data. In addition, user acceptability, long-term stability, and postmarket outcomes also need further evaluation. Future research directions include clinical trials, enhanced formulations, user-focused adaptations, and sustainable manufacturing practices. These steps will help to validate the efficacy of YOSHA and support its integration into modern female hygiene care.

### Dissemination Plan

This protocol will be published in the form of a thesis to share our research on Ayurvedic intimate wash. The thesis will provide a thorough overview of the study’s design, methodology, data gathering methods, data analysis plan, and ethical considerations. We hope that by making this protocol public, we will contribute to expanding knowledge in the field and promoting future research efforts.

### Conclusions

The study will show that YOSHA, a modern herbal product based on the traditional Ayurvedic yoniprakshalan kwatha, has strong in vitro antimicrobial effects against common vaginal pathogens and meets the pharmaceutical quality and safety standards. Unlike raw decoctions, it offers a stable, user-friendly formulation that blends traditional knowledge with scientific validation. Strengths include classical preparation methods, solid quality control, and reliable results. Future work should involve clinical trials, broader testing, and user-focused evaluations. Findings will be shared through journals, conferences, and practitioner outreach to promote YOSHA as a natural option for women’s hygiene.

## Abbreviations:

CRIS: Checklist for Reporting In-Vitro Studies
MGACHRC: Mahatma Gandhi Ayurved College, Hospital and Research Centre
NCISM: National Commission for Indian Systems of Medicine
